# Role of Caveolin 1, E-Cadherin, Enolase 2 and PKCalpha on resistance to methotrexate in human HT29 colon cancer cells

**DOI:** 10.1186/1755-8794-1-35

**Published:** 2008-08-11

**Authors:** Elisabet Selga, Cristina Morales, Véronique Noé, Miguel A Peinado, Carlos J Ciudad

**Affiliations:** 1 Department of Biochemistry and Molecular Biology, School of Pharmacy, University of Barcelona, Barcelona, Spain; 2 Institut d'Investigació Biomèdica de Bellvitge (IDIBELL), L'Hospitalet, Barcelona, Spain; 3 Institut de Medicina Predictiva i Personalitzada del Càncer (IMPPC), Badalona, Barcelona, Spain; 4 Biotech Research and Innovation Centre (BRIC), University of, Copenhagen, Ole Maaløes Vej 5, DK-2200 Copenhagen, Denmark

## Abstract

**Background:**

Methotrexate is one of the earliest cytotoxic drugs used in cancer therapy, and despite the isolation of multiple other folate antagonists, methotrexate maintains its significant role as a treatment for different types of cancer and other disorders. The usefulness of treatment with methotrexate is limited by the development of drug resistance, which may be acquired through different ways. To get insights into the mechanisms associated with drug resistance and sensitization we performed a functional analysis of genes deregulated in methotrexate resistant cells, either due to its co-amplification with the *dhfr *gene or as a result of a transcriptome screening using microarrays.

**Methods:**

Gene expression levels were compared between triplicate samples from either HT29 sensitive cells and resistant to 10^-5 ^M MTX by hybridization to the GeneChip^® ^HG U133 PLUS 2.0 from Affymetrix. After normalization, a list of 3-fold differentially expressed genes with a p-value < 0.05 including multiple testing correction (Benjamini and Hochberg false discovery rate) was generated. RT-Real-time PCR was used to validate the expression levels of selected genes and copy-number was determined by qPCR. Functional validations were performed either by siRNAs or by transfection of an expression plasmid.

**Results:**

Genes adjacent to the *dhfr locus *and included in the 5q14 amplicon were overexpressed in HT29 MTX-resistant cells. Treatment with siRNAs against those genes caused a slight reduction in cell viability in both HT29 sensitive and resistant cells. On the other hand, microarray analysis of HT29 and HT29 MTX resistant cells unveiled overexpression of caveolin 1, enolase 2 and PKCα genes in resistant cells without concomitant copy number gain. siRNAs against these three genes effectively reduced cell viability and caused a decreased MTX resistance capacity. Moreover, overexpression of E-cadherin, which was found underexpressed in MTX-resistant cells, also sensitized the cells toward the chemotherapeutic agent. Combined treatments targeting siRNA inhibition of caveolin 1 and overexpression of E-cadherin markedly reduced cell viability in both sensitive and MTX-resistant HT29 cells.

**Conclusion:**

We provide functional evidences indicating that caveolin 1 and E-cadherin, deregulated in MTX resistant cells, may play a critical role in cell survival and may constitute potential targets for coadjuvant therapy.

## Background

Colorectal cancer is the third most common form of cancer and the second leading cause of cancer-related death in the Western world. Colon cancer causes 655,000 deaths worldwide per year [1]. Therapy is usually through surgery, followed in many cases by chemotherapy, which is used to slow tumor growth, to shrink tumor size and to reduce the likelihood of metastasis development.

Chemotherapy effectiveness in cancer cells is compromised by the achievement of drug resistance. Therefore, gaining insight into the mechanisms underlying drug resistance is basic to develop more effective therapeutic approaches. Morales *et al*. [2] hypothesized that the genetic features related with the progression pathway in colorectal cancer may condition its chemoresistance capability. In fact, it has been described that the tumor's ability to survive, grow and metastasize is conditioned by its genetic and phenotypic heterogeneity [3].

Methotrexate (MTX) is an antimetabolite and antifolate drug used in treatment of cancer and autoimmune diseases. MTX competitively and reversibly inhibits dihydrofolate reductase (DHFR), an enzyme that participates in folate metabolism, and essential for DNA synthesis and cell growth [4]. MTX is used for the treatment of lymphoblastic leukemia, lymphoma, osteosarcoma, breast cancer, and head and neck cancer [5]. Treatments combining MTX and other drugs are used in colorectal cancer [6-8]. However, MTX resistance can be easily acquired through different ways, although amplification of the target gene (*dhfr*) has been shown to be the most important mechanism of resistance in cultured cells [9-11]. Indeed, amplification of 5q12-14 regions, where *dhfr *is located, has been described in MTX-resistant HT29 cells [2].

In the present study, we wanted to identify genes implicated in MTX resistance in HT29 colon cancer cells and to explore their relative contribution to this phenotype. We analyzed the differential gene expression between MTX-resistant and MTX-sensitive HT29 cells using oligonucleotide microarrays containing the full human genome. Changes in the DNA content between both cell lines were also determined. We showed a role for specific differentially expressed genes in MTX resistance. Using siRNAs against caveolin 1, enolase 2 and PKCα or plasmid overexpression for E-cadherin, a clear chemosensitization toward MTX was observed.

## Methods

### Cell Culture

Human colon adenocarcinoma cell line HT29 was routinely grown in Ham's F12 medium supplemented with 7% fetal bovine serum (FBS, both from Gibco) at 37°C in a 5% CO_2 _humidified atmosphere. Cells resistant to 10^-5 ^M MTX, which corresponds to a 1000-fold increase in resistance with respect to the sensitive cells, were previously obtained in the laboratory [12] upon incubation with stepwise concentrations of MTX (Lederle) and were rutinely grown in selective DHFR medium (-GHT medium, GIBCO) lacking glycine, hypoxanthine and thymidine, the final products of DHFR activity. This medium was supplemented with 7% dialyzed fetal bovine serum (GIBCO).

### Microarrays

Gene expression was analyzed by hybridization to the GeneChip^® ^Human Genome U133 PLUS 2.0 from Affymetrix, containing over 47,000 transcripts and variants. Total RNA for oligo arrays was prepared from triplicate samples of both HT29 sensitive and resistant cells using the RNAeasy Mini kit (Qiagen) following the recommendations of the manufacturer. Labeling, hybridization and detection were carried out following the manufacturer's specifications. The data discussed in this publication have been deposited in NCBIs Gene Expression Omnibus [13] and are accessible through GEO Series accession number GSE11440.

### Microarray data analysis

Quantification was carried out with GeneSpring GX software v 7.3.1 (Silicon Genetics), which allows multi-filter comparisons using data from different experiments to perform the normalization, generation of restriction lists and functional classifications of the differentially expressed genes. Normalization was applied in two steps: i) "per Chip normalization" by which each measurement was divided by the 50th percentile of all measurements in its array; and ii) "per Gene normalization" by which all the samples were normalized against the median of the control samples (HT29 sensitive cells). The expression of each gene was reported as the ratio of the value obtained after each condition relative to the control condition after normalization of the data. Then, data were filtered using the control strength, a control value calculated using the Cross-Gene Error Model on replicates [14] and based on average base/proportional value. Measurements with higher control strength are relatively more precise than measurements with lower control strength. Genes that did not reach this value were discarded. Additional filtering was performed to determine differentially expressed genes. A restriction t-test p-value of less than 0.05 including multiple testing correction (Benjamini and Hochberg false discovery rate) was applied. The output of this analysis was then filtered by fold expression, to specifically select those genes that had a differential expression of at least 3-fold. The 375 transcripts included in this list can be viewed in Additional file 1.

### RT-Real-Time PCR

mRNA levels of the different selected genes were determined by RT-Real-time PCR. Total RNA was extracted from cells (4 × 10^6^) using Ultraspec™ RNA reagent (Biotecx) following the recommendations of the manufacturer. Complementary DNA was synthesized in a total volume of 20 μl from RNA samples by mixing 500 ng of total RNA, 125 ng of random hexamers (Roche), in the presence of 75 mM KCl, 3 mM MgCl_2_, 10 mM dithiothreitol, 20 units of RNasin (Promega), 0.5 mM dNTPs (AppliChem), 200 units of M-MLV reverse transcriptase (Invitrogen) and 50 mM Tris-HCl buffer, pH 8.3. The reaction mixture was incubated at 37°C for 60 min and the cDNA product was used for subsequent Real-time PCR amplification using SYBR Green. A standard 20 μl reaction contained 25 ng of the cDNA mixture, 0.5 μM of the forward and reverse primers and the SYBR Green Master Mix. Primers used are listed in the Additional file 2.

### Determination of gene copy number

Genomic DNA from either HT29 sensitive or resistant cells was obtained with the Wizard™ Genomic DNA Purification Kit (Promega) following the manufacturer's recommendations. Five nanograms of DNA were used for Real-Time PCR amplification in a 20 μl reaction containing 0.5 μM of the forward and reverse primers and the SYBR Green Master Mix in an ABI Prism 7000 Sequence Detection System (Applied Biosystems). A list of the primers used is provided as Additional file 3.

### Functional validations

#### A) transfection of siRNAs against selected genes

HT29 cells (30,000) were plated in 1 ml of -GHT medium and transfection was performed eighteen hours later. For each well, 4 μl of Lipofectamine™ 2000 (Invitrogen) in 100 μl of serum free -GHT medium were mixed in Eppendorf tubes with 100 nM of siRNA in 100 μl of serum free -GHT medium. The mixture was incubated at room temperature for 20 min before addition to the cells. MTX (5 × 10^-8 ^M) was added 48 hours after siRNA treatment and MTT assays were performed [15] after 5 days from the beginning of the treatment. Treatment of HT29 resistant cells was performed following the same protocol using 2 μl of Lipofectamine™ 2000 and 10^-5 ^M MTX. When screening for mRNA levels of the different genes after siRNA treatment, 30,000 cells, either sensitive or resistant, were incubated with increasing amounts of siRNA (1–100 nM) maintaining a 3:1 ratio (μl of Lipofectamine : μg siRNA) and following the procedure previously described. Cells were harvested 48 hours after siRNA treatment for RNA extraction and RT-Real-time PCR. In the combination experiments with siRNAs, 100 nM of each siRNA were diluted in the same eppendorf containing 100 μl of serum free -GHT medium and combined with Lipofectamine™ 2000 as described above. MTX was added as in the single siRNA experiments, mRNA levels were determined and MTT was performed as previously described. In all cases, a non-related siRNA was used as negative control. The treatment was performed as described above and cell viability and mRNA levels for each gene were quantified in parallel. The siRNAs were designed using the software iRNAi. Then, BLAST resources in NCBI were used to assess the degree of specificity of the sequence recognition for these siRNAs. We only selected the siRNAs that reported the target gene as the only mRNA hit. The sequences for the sense strand of all siRNAs used are available in the supplementary material provided (see Additional file 4).

#### B) transfection of an expression plasmid encoding for E-cadherin

HT29 cells were seeded into 6-well plates at a density of 3 × 10^4 ^cells/well in 1 ml of HAM F12 selective medium. Eighteen hours later, transfections with the expression plasmid for E-cadherin (pBATEM2-CDH) were performed in the presence or in the absence of MTX. The overexpression of E-cadherin was monitored by determining its mRNA levels after 48 h upon transfection. For each well, Lipofectamine™ 2000 was diluted in 100 μl of serum free -GHT medium and was combined with different amounts of the plasmid (500 ng-5 μg) in 100 μl of serum free -GHT medium, always maintaining a 2:1 ratio (μl of Lipofectamine : μg of plasmid). After 20 min at room temperature, the mixture was added to the cells. When combining pBATEM2-CDH transfection and MTX treatment, 5 × 10^-8 ^M MTX was added 48 h after transfection. Cell viability was measured by the MTT assay after 5 days from the beginning of the treatment. Treatment of HT29 resistant cells was performed following the same steps but using 10^-5 ^M MTX.

#### C) co-transfection of siCAV1 and pBATEM2-CDH

When transfection of siCAV1 and pBATEM2-CDH was performed simultaneously, 100 nM of siRNA and 1 μg of plasmid were diluted together in Eppendorf tubes with 100 μl of serum free -GHT medium and mixed with lipofectamine™ 2000 in 100 μl of serum free -GHT medium (6 μl for the sensitive cells and 3 μl for the MTX-resistant cells). The mixture was incubated at room temperature for 20 min before addition to the cells (3 × 10^4 ^cells/well in 1 ml of HAM F12 selective medium, pre-seeded eighteen hours earlier). The mRNA levels after transfection were determined for both genes as previously described and MTT assay was used to determine cell viability.

## Results

### Identification of genes deregulated in association with MTX resistance

**Figure 1:**
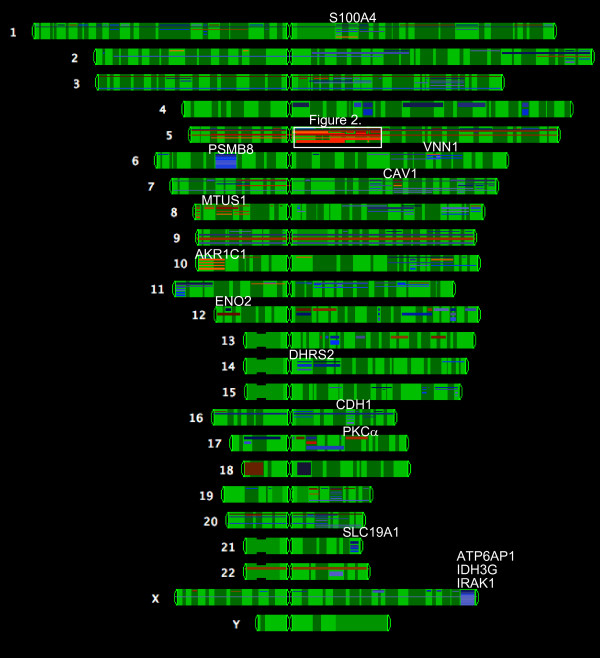
**Chromosomal view of the differentially expressed genes in HT29 MTX-resistant cells**. The expression values of genes included in the 3-fold differentially expressed list were viewed in their respective chromosomal location. The names for all the genes studied are depicted on top of their chromosome position. Red is used to color the overexpressed genes and blue is used to highlight the underexpressed genes.

**Figure 2:**
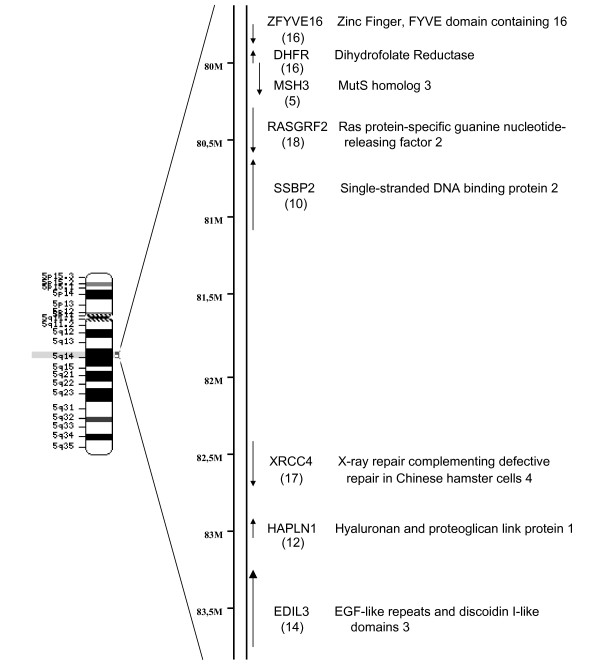
**Localization of *dhfr *and other genes in chromosome 5 that are overexpressed in HT29 MTX-resistant cells**. It is presented a magnification of the region in chromosome 5 where *dhfr *is located (5q14). The left part is an ideogram of chromosome 5; the right part shows the relative position of all genes studied that are located in this chromosome and that were amplified. The arrows indicate their transcription orientation and the values in parentheses under the names correspond to their respective copy-number validated by Real-time PCR.

The expression profile of the 47,000 transcripts and variants included in the HG U133 PLUS 2.0 microarray from Affymetrix was compared between HT29 sensitive cells and resistant to 10^-5 ^M MTX. GeneSpring GX software v7.3.1 was used to analyze the results. A list of 3-fold differentially expressed genes was generated as described in Methods (Additional file 1). The expression values for genes in this list can be viewed in their corresponding chromosomal position (Figure 1). This overlapping view evidenced a highly overexpressed region in chromosome 5 that covers *dhfr *and the surrounding *loci*. The set of upregulated genes in this location included *dhfr*, *zfyve16*, *msh3*, *rasgrf2*, *ssbp2*, *xrcc4*, *hapln1 *and *edil3 *(Figure 2), which were selected for further studies. Additional genes that were clearly overexpressed or underexpressed and located in other human chromosomes were also selected according to their function and after literature mining of genes related to drug resistance. The expression levels of most of the selected genes were validated by RT-Real-time PCR (Table 1). The correlation between microarray and qPCR was calculated using the log-transformed values of the fold change obtained for the selected genes, obtaining an r-value of 0.95. To test if changes in the DNA content were responsible for the expression levels of the selected genes in the resistant cells, we determined the copy number for all of them using Real-Time PCR. The results, presented in table 1, showed amplification of all the genes in chromosome 5 flanking *dhfr*, as well as of *mtus1*, located in chromosome 8. *E-cadherin *was the only gene clearly lost among the selected genes.

**Table 1:** mRNA levels and copy number determination of differentially expressed genes in HT29 MTX-resistant cells.

**GenBank**	**Gene Name**	**Chromosome**	**Copy Number (Q-PCR)**	**Expression**		**Gene Function**
				**Microarrays**	**Validation (RT-PCR)**	
NM_002961	S100A4	1	0.85 ± 0.1	3.7 (p = 5.5e^-6^)	5.68 ± 0.4	Angiogenesis
BU078629	ZFYVE16	5	16.81 ± 2.1	6.1 (p = 7.7e^-6^)	6.7 ± < 0.1	Zinc ion binding
AI144299	DHFR	5	16.09 ± 1.4	7.1 (p = 1.2e^-7^)	11.05 ± 0.5	Nucleotide metabolism
NM_002439	MSH3	5	4.97 ± 0.5	3.9 (p = 5.5e^-6^)	4.23 ± 0.4	Missmatch repair
AI912976	RASGRF2	5	17.76 ± 0.4	4.6(p = 8.9e^-5^)	6.10 ± 0.5	MAPK signaling
AF912976	SSBP2	5	10.27 ± 0.7	2.4 (p = 3.4e^-3^)	2.96 ± 0.2	ss DNA binding
NM_022406	XRCC4	5	17.31 ± 1.1	7.1 (p = 4.7e^-6^)	8.90 ± 2.3	ds break repair
U43328	HAPLN1	5	11.55 ± < 0.1	147 (p = 2.9e^-10^)	1111.9 ± 80.7	Cell adhesion
AA053711	EDIL3	5	14.3 ± 0.7	157 (p = 9.1e^-8^)	N/D	Cell adhesion
U17496	PSMB8	6	0.91 ± < 0.1	0.1 (p = 0.01)	N/D	Proteasome subunit
NM_004666	VNN1	6	0.84 ± < 0.1	0.04 (p = 0.01)	N/D	Nitrogen metabolism
AU147399	CAV1	7	1.14 ± < 0.1	10.9 (p = 1.5e^-4^)	15.00 ± 0.8	Integ. plasma membr.
BE552421	MTUS1	8	3.52 ± 0.1	3.4 (p = 1.8e^-6^)	N/D	Mitoc. tumor suppressor
U05598	AKR1C1	10	0.94 ± < 0.1	4,6 (p = 3.9e^-6^)	6.72 ± 0.7	Xenobiotics metabolism
NM_001975	ENO2	12	0.92 ± < 0.1	6.0 (p = 4.6e^-6^)	3.90 ± 0.1	Glycolisis
AK000345	DHRS2	14	0.97 ± < 0.1	0.12 (p = 0.01)	N/D	Oxidoreductase
L08599	CDH1	16	0.33 ± < 0.1	0.19 (p = 0.01)	0.15 ± < 0.1	Cell adhesion
AI471375	PRKCA	17	1.05 ± < 0.1	4.2 (p = 1.7e^-5^)	2.55 ± 0.2	Regulation cell cycle
BQ003811	SLC19A1	21	0.84 ± < 0.1	0.1 (p = 0.01)	N/D	Cell adhesion
NM_001569	IRAK1	X	1.25 ± < 0.1	0.26 (p = 7.3e^-3^)	N/D	IL1 receptor Kinase
NM_004135	IDH3G	X	0.85 ± < 0.1	0.28 (p = 7.3e^-8^)	N/D	TCA cycle
NM_001183	ATP6AP1	X	0.68 ± < 0.1	0.3 (p = 0.01)	N/D	ATP biosynthesis

Twenty-two genes belonging to the 3-fold differentially expressed list were selected according to their possible relation with drug resistance and/or chromosomal localization. It is shown the GenBank accession number of all genes next to their common name, and their chromosome number. Real-Time PCR was used to determine their copy number, and the expression levels for all them are presented both as the values found in the microarrays (in fold changes relative to the control; t-test p-values included) and as validated mRNA levels (using RT-Real-Time PCR). All experimental results are expressed as fold changes referred to the sensitive cells and values are the mean of triplicate experiments ± SE. The last column indicates the functional categories of the genes. N/D, nondetermined value.

### Effect on MTX sensitivity of siRNAs against genes flanking *dhfr*

**Figure 3:**
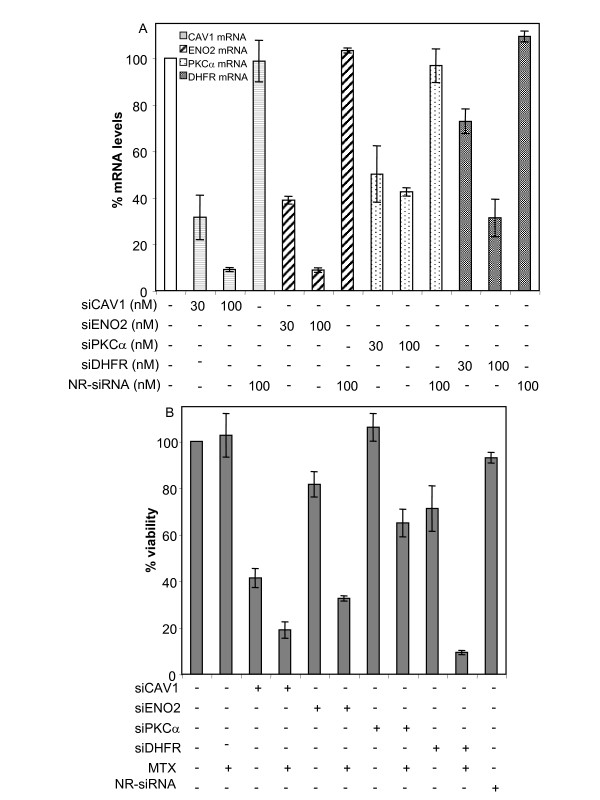
**Determination of mRNA levels and cytotoxicity upon siRNA treatment of HT29 sensitive cells**. (A) The mRNA levels of CAV1, ENO2, PKCα and DHFR were determined by RT-Real-time PCR 48 h after treatment of HT29 cells with the indicated concentrations of siCAV1, siENO2, siPKCα, siDHFR and a non-related siRNA. Symbols used for each mRNA are presented as an insert within the figure. (B) Cells were treated with 100 nM of each siRNA as previously described and 5 × 10^-8 ^M MTX was added after 48 h. Cell viability was determined after 5 days from the beginning of the treatment. All results are expressed as percentages referred to untreated cells. Values are the mean of three independent experiments ± SE. A non-related (NR) siRNA was used as negative control.

To investigate if the genes that were both overexpressed and co-amplified with *dhfr *contributed to MTX resistance, their mRNA levels were brought down by means of siRNAs. Effective reduction (≈ 70%) of the respective mRNAs was obtained upon transfection of 100 nM of each single siRNA, both in sensitive and resistant cells. These treatments, though, caused a small reduction in the viability of both cell lines, and addition of MTX to the siRNAs did not sensitize the cells toward the chemotherapeutic agent (data not shown). On the contrary, a siRNA against *dhfr *mRNA (siDHFR) caused a reduction in cell viability of 30% in HT29 sensitive cells, which was increased up to 90% with the addition of MTX (Figure 3B). mRNA levels after siDHFR treatment were reduced by 70% in this cell line (Figure 3A).

### Effect on MTX sensitivity of siRNAs against CAV1, ENO2, PKCα and DHFR

**Figure 4:**
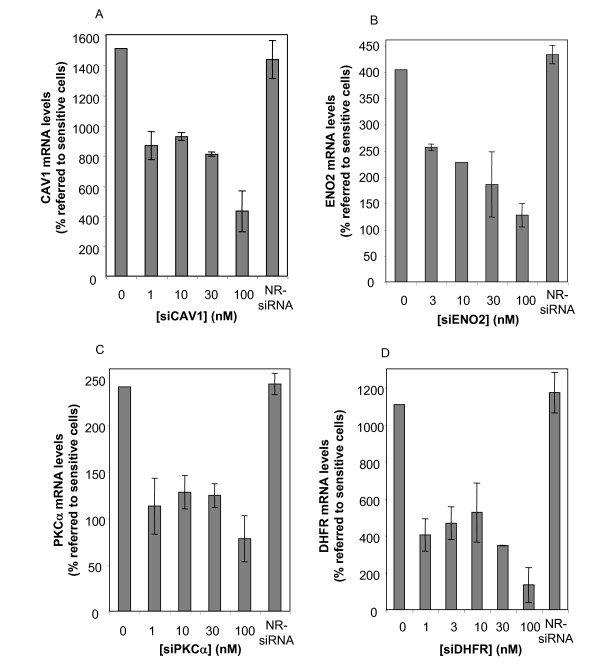
**mRNA levels of CAV1, ENO2, PKCα and DHFR upon siRNA treatment of MTX-resistant cells**. Treatments with increasing amounts of each siRNA were performed in MTX-resistant HT29 cells. Forty-eight hours later, mRNA levels for each gene were determined and expressed as percentages of the untreated control (A, CAV1; B, ENO2; C, PKCα and D, DHFR). A non-related (NR) siRNA was used as negative control. Results are depicted taking into account the relative gene expression in the resistant cells (% of the sensitive cells). Values are the mean of three independent experiments ± SE.

As the knocking down of genes co-amplified with *dhfr *showed only a slight contribution to MTX sensitivity, we focused in three genes that were clearly overexpressed in the resistant cells and located in different chromosomes, namely caveolin 1(CAV1), enolase 2 (ENO2) and PKCα. We quantified the mRNA levels of these three genes after treatment with different concentrations of the corresponding siRNA using RT-Real-time PCR. The three siRNAs were effective in reducing the mRNA levels of their targets, both in sensitive (Figure 3A) and in MTX-resistant (Figure 4) HT29 cells. One hundred nanomolar was the most effective concentration for all of them, and was used in subsequent experiments. The mRNA levels upon treatment with the siRNA against DHFR are also presented in these series (Figure 3A &4). The mRNA levels of the four genes after treatment with their respective siRNAs in the resistant cells were reduced down almost to the expression levels found for these genes in HT29 sensitive cells (compare Y-axes between Figure 3A &4). A non-related siRNA was used as negative control, and did not produce any significant reduction on the mRNA levels of any of the four genes, either in sensitive or in resistant HT29 cells.

Viability of HT29 sensitive cells (Figure 3B) was moderately reduced upon treatment with 100 nM siENO2 or siDHFR and treatment with siCAV1 caused a marked reduction of cell viability on its own. No effect on cell viability was observed upon treatment with siPKCα. In all cases, treatment with 100 nM of each single siRNA increased the sensitivity of HT29 cells toward MTX with respect to the control: 80% when using siCAV1; 70% with siENO2; 40% with siPKCα and 90% when siDHFR was used. However, when the same treatments were performed in MTX-resistant cells (data not shown), cell viability was reduced only by 15% when using either siCAV1, siENO2 or siPKCα, and by 25% when siDHFR was used. None of these effects were improved by the combination of siRNAs with MTX. Transfection with 100 nM of a non-related siRNA did not cause any significant reduction on cell viability, either in sensitive or in resistant HT29 cells.

### Effect of the combination of siRNAs against CAV1, ENO2, PKCα and DHFR on MTX sensitivity

**Figure 5:**
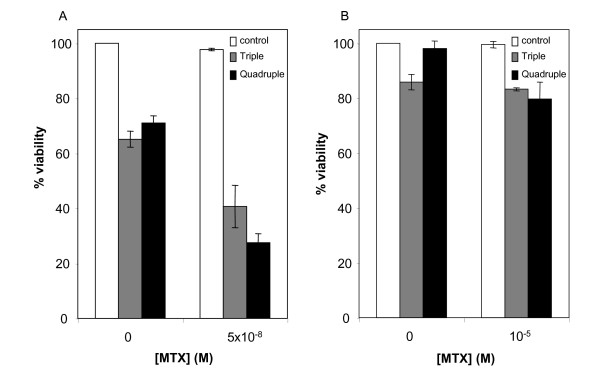
**Effects of combining siRNAs against CAV1, ENO2, PKCα and DHFR on MTX sensitivity**. Treatments with combinations of siRNAs at100 nM each were performed both in sensitive (A) and in resistant cells (B). MTX was added after 48 h and cell viability was determined by the MTT assay after 5 days from the beginning of the treatment. The triple combination includes the siRNAs against CAV1, ENO2 and PKCα; the quadruple combination includes the three previous siRNAs plus siDHFR. Results are presented as percentages referred to the untreated cells. Values are the mean of three independent experiments ± SE.

As we had observed a chemosensitization toward MTX in sensitive cells when using individual siRNAs, we performed experiments including the siRNAs against CAV1, ENO2 and PKCα (triple combination) or in combination with siDHFR (quadruple combination) to test if these combinations also increased the sensitivity toward MTX. Treatments combining the three siRNAs (siCAV1, siENO2 and siPKCα) at 100 nM each reduced cell viability by 30% and effectively increased MTX sensitivity by 60% with respect to the control in HT29 sensitive cells (Figure 5A). Addition of 100 nM siDHFR to the previous combination caused a reduction on cell viability of the same degree as the triple combination but increased MTX sensitivity to about 75%. In the case of MTX-resistant HT29 cells, treatments were performed with the same combinations (Figure 5B). The triple combination reduced cell viability by 15% on its own. However, MTX sensitivity was not improved. The quadruple combination did not affect cell viability on its own but caused a reduction of 20% on cell viability when combined with MTX. It was confirmed that the mRNA levels of the four genes were decreased after the siRNA combination treatments in both cell lines (Table 2). Treatments with a non-related siRNA at 400 nM were performed in order to assess the citotoxicity of triple and quadruple combinations and to verify the mRNA levels of all four genes. No effect was observed in any case in either sensitive or resistant cells.

**Table 2:** mRNA levels upon treatment with combination of siRNAs against CAV1, ENO2, PKCα and DHFR.

A				
Treatment	CAV1	ENO2	PKCα	DHFR

siCAV1 + siENO2 + siPKCα	50.1 ± 4.3	50.5 ± 4.4	66.2 ± 2.6	82.9 ± 2.3
siCAV1 + siENO2 + siPKCα + siDHFR	37.7 ± 3.7	59.1 ± 0.8	47.6 ± 4.7	47.3 ± 1.2
400 nM NR-siRNA	96.8 ± 10.4	98.4 ± 0.5	96.3 ± 0.3	100.6 ± 13.4

B				

Treatment	CAV1	ENO2	PKCα	DHFR

siCAV1 + siENO2 + siPKCα	62.7 ± 0.2	44.20 ± 0.9	73.2 ± 4.9	97.7 ± 8.8
siCAV1 + siENO2 + siPKCα + siDHFR	45.4 ± 0.8	36.34 ± 4.2	40.6 ± 2.5	33.4 ± 3.8
400 nM NR-siRNA	99.1 ± 9.2	100.57 ± 15.9	103.2 ± 1.1	95.5 ± 10.2

Treatments combining the siRNAs against CAV1, ENO2 and PKCα at 100 nM each were performed both in sensitive (A) and in resistant (B) HT29 cells. The mRNA levels for all three genes were determined at 48 hours of treatment. DHFR mRNA levels were also determined. Treatments combining 100 nM of the previous three siRNAs plus a siRNA against DHFR were also performed in both cell lines and mRNA levels for the four genes quantified by RT-Real-Time PCR. A non-related (NR) siRNA was used as negative control. Results are expressed as percentages of mRNA referred to untreated cells (mean ± SE) of triplicate experiments.

### Effect of overexpressing E-cadherin on its mRNA levels, cell viability and MTX sensitivity

**Figure 6:**
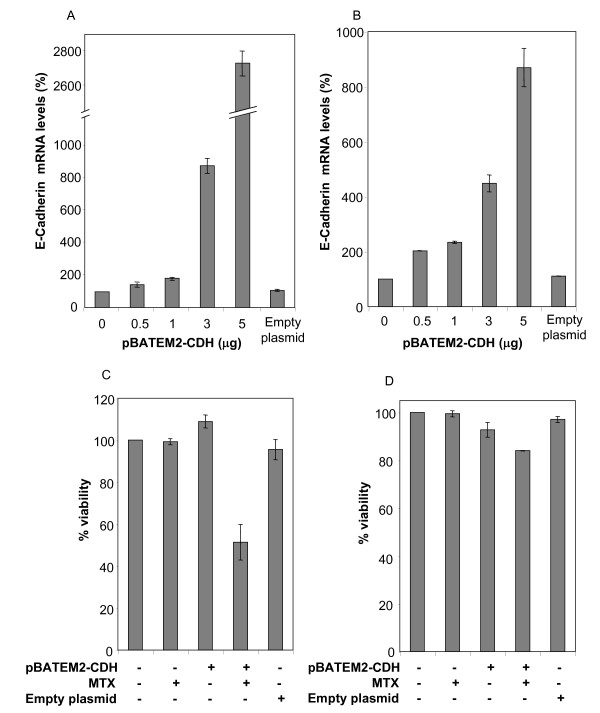
**Determination of mRNA levels and cytotoxicity upon overexpression of E-cadherin**. Cells were treated with increasing amounts of an expression plasmid encoding for E-cadherin (pBATEM2-CDH). Forty-eight hours after the treatment, mRNA levels were determined in HT29 sensitive (A) and MTX-resistant cells (B). Three independent experiments were performed and results are expressed as percentages referred to untreated cells. Values are the mean ± SE. Simultaneous experiments of cells transfected with pBATEM2-CDH were treated with MTX 48 h after transfection, and viability of both sensitive (C) and MTX-resistant cells (D) was assessed after 5 days from the beginning of the treatment by the MTT assay. The mean value ± SE of three independent experiments is depicted. An empty plasmid was used as negative control both for mRNA levels and cytotoxicity determination.

Since E-cadherin was lost and underexpressed in the resistant cells, it was transiently expressed in HT29 sensitive and resistant cells by means of an expression vector (pBATEM2-CDH). Cells were harvested after 48 hours of treatment. RT-Real-Time PCR was used to quantify E-cadherin mRNA levels in both cell lines (Figure 6A &6B). Transfection of more than 1 μg of the expression vector caused a marked reduction on cell viability in both cell lines. Therefore, 1 μg of plasmid was used in all subsequent experiments. Overexpression of E-cadherin was performed in HT29 sensitive cells in the absence or in the presence of 5 × 10^-8 ^M MTX. This treatment increased by 50% the effect of methotrexate (Figure 6C), thus providing evidence that loss of E-cadherin can confer increased resistance of HT29 cells toward MTX. The same approach was used with HT29 resistant cells, in combination or not with 10^-5 ^M MTX. Overexpression of E-cadherin reduced by 10% cell viability of the resistant cells and only a small improvement was observed when combining E-cadherin overexpression with MTX treatment (Figure 6D).

### Effect of co-transfection of siCAV1 and pBATEM2-CDH on MTX sensitivity

**Figure 7:**
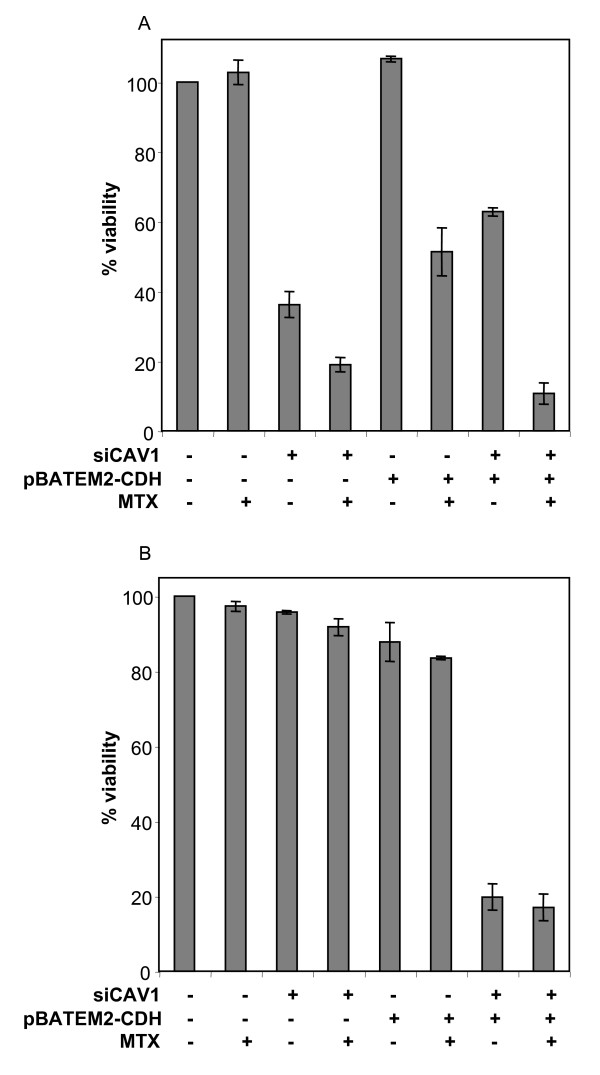
**Effect of combining the siRNA against CAV1 and the expression plasmid for E-cadherin**. One hundred nanomolar siRNA against CAV1 and 1 μg of the expression plasmid for E-cadherin were transfected in both sensitive (A) and resistant (B) HT29 cells. MTX was added 48 hours after transfection and the MTT assay was used to determine cell viability. Results are expressed as percentages referred to untreated cells. Values are the mean of three independent experiments ± SE.

E-cadherin has been shown to be an important permissive element in defining the functions of CAV1 [16]. Thus, we performed co-transfection experiments to reduce the mRNA levels of CAV1 and to overexpress E-cadherin simultaneously. mRNA levels after co-transfection were determined in both cell lines (Table 3). As observed in figure 7A, altering the mRNA levels for the two genes reduced the viability of HT29 sensitive cells by almost 40%. Moreover, addition of MTX to the previous combination further reduced cell viability by 90%. Importantly, when performing these co-transfection experiments in HT29 resistant cells, cell viability was reduced by 80%, although in this instance MTX did not improve the effect (Figure 7B).

**Table 3:** mRNA levels upon treatment with siCAV1 and pBATEM2-CDH.

Treatment	Cell line	CAV1	E-cadherin
siCAV1 + pBATEM2-CDH	HT29 sensitive	21.8 ± 1.9	252.3 ± 3.5
siCAV1 + pBATEM2-CDH	HT29 resistant	25.6 ± 0.2	199.7 ± 16.9

Transfection experiments combining the siRNA against caveolin 1 (siCAV1) and the expression plasmid for E-cadherin (pBATEM2-CDH) were performed both in HT29 sensitive and MTX-resistant cells. Forty-eight hours later, mRNA levels for the two genes were determined in both cell lines by RT-Real-Time RCR. Results are expressed as percentages of mRNA referred to untreated cells (mean ± SE) of at least 3 independent experiments.

## Discussion

In the present study, genes differentially expressed in HT29 colon cancer cells resistant to MTX were identified and their relative contribution to this phenotype evaluated. We observed a cluster of genes flanking the *dhfr locus *in chromosome 5 that were overexpressed in MTX-resistant HT29 cells. Two of the genes included in this cluster, MSH3 and XRCC4, are known to be involved in DNA repair [17-19]; other two, RASGRF2 and SSBP2, have been related to signaling pathways [20-22]; and EDIL3 has been suggested to prevent apoptosis and to promote cell proliferation [23,24]. Despite the confirmation of the co-amplification of all these genes with *dhfr *in the resistant cells, we did not observe a clear sensitization toward MTX when reducing their respective mRNA levels by means of iRNA technology. Our observations indicate that the increase in copy-number and the resulting upregulation of the studied genes in 5q14 may be a consequence of *dhfr *amplification more than an adaptation of the cells to MTX resistance. Indeed, many mammalian species (mouse, rat, bull, cock, dog and chimpanzee) show this set of genes in the same order around *dhfr *as in human chromosome 5 (using the MapViewer at NCBI), indicating a conserved pattern of gene organization. In keeping with this, its overexpression in the resistant cells could have been useful to improve some cellular processes that might facilitate survival. However, as shown in this work, the increase in copy number of this set of genes does not favor MTX resistance. Thus, we decided to search for other differentially expressed genes (CAV1, E-cadherin, ENO2 and PKCα) that had been previously related with resistance or with colon cancer and to evaluate their relative contribution in our cell system.

Enolase 2 (ENO2) is induced by hypoxia, an intrinsic condition of tumors. Moreover, ENO2 is a glycolysis-related gene that has been described to play an important role in tumorogenesis of colorectal cancers [25]. Indeed, ENO2 is upregulated in a variety of cancers [26-28] and alpha-enolase is significantly upregulated in a metastasic colon cancer cell line, suggesting a possible association with the metastasic process *in vitro *and *in vivo *[29]. Indeed, we observed a notable contribution of ENO2 to MTX resistance when treating the sensitive cells with siENO2.

Both the α-isozyme of PKC and caveolin 1 has been described to be associated with multidrug resistance [30,31], and thus represent good targets to be analyzed. PKCα phosphorylates different proteins, which triggers a wide variety of cellular responses including proliferation, differentiation, membrane transport, gene expression and tumor promotion [32,33]. Chemical inhibitors of PKC activity have been proposed as resistance modulators in MTX chemotherapy [34]. Furthermore, decreasing PKCα mRNA levels attenuates the MDR phenotype in tumor cells [35] and increases the sensitivity to anticancer drugs, both *in vitro *[36-38] and *in vivo *[39]. These observations are in accordance with our result showing that the decrease of PKCα mRNA levels by means of iRNA technology causes a sensitization of the cells toward MTX. Caveolin 1 (CAV1), the principal component of caveolae, has been associated with progression of colon and breast carcinomas [40,41] and with enhanced invasiveness in lung adenocarcinoma cells [42]. Although suggested as tumor suppressor gene, and downregulated in some oncogene-transformed and tumor-derived cells [31], overexpression of CAV1 has been found in prostate and esophageal cancer [43-45]. Moreover, re-expression of CAV1 at latter stages of tumor development has been described in human and mouse prostate adenocarcinomas [41], a scenario that could resemble chemotherapy resistance. Indeed, Bender *et al*. [46] found significantly higher levels of CAV1 in MTX resistant HT29 clones. We have confirmed the implication of CAV1 in MTX resistance in our HT29 cell line.

Nevertheless, as Benimetskaya and collaborators observed with PKCα [47], downregulation of a gene alone may be insufficient to completely chemosensitize the cells. Therefore, we considered a combination therapy in order to improve MTX sensitivity. As shown in figure 5, the combination of siRNAs against CAV1, ENO2 and PKCα sensitizes the cells toward MTX, and the effect is improved by the additional downregulation of DHFR. The effects of the triple or the quadruple combinations, however, are not the sum of the effects caused by each single siRNA. This probably reflects the difficulty of transfecting more than one siRNA at 100 nM each. Indeed, the mRNA levels for the four genes after the combination treatment were not as reduced as with the single treatments. In the case of all treatments performed in the resistant cells, probably the overexpression by amplification of the *dhfr locus *was powerful enough to mask the effects of the siRNAs used.

Not only the overexpression of some genes may cause the resistance phenotype. One of the most underexpressed genes that we confirmed to be clearly lost in our HT29 MTX-resistant cells is *E-cadherin*. In fact, loss of *E-cadherin*, frequently observed in epithelial tumors, has been associated with tumor progression [48,49] and is considered a crucial event that favors metastasis and invasiveness [50,51]. In addition, the mRNA levels of E-cadherin in adenocarcinoma are 2-fold lower than in normal colon cells [52]. Thus, there is a functional correlation between E-cadherin levels and malignancy. It has been described an event of loss of heterozygosity at the 16.1q chromosome band in most human prostate cancers, where *E-cadherin *is located [53], which is associated with tumor grade, advanced clinical stage and poor survival [54]. Our experiments show a decrease of 3-fold in E-cadherin levels in resistant cells and also that a mild overexpression of E-cadherin causes a higher sensitivity toward MTX. One has to be cautious, however, about the expression levels of E-cadherin since an increase of more than 3-fold in any of both cell lines caused a reduction in cell viability. This is in accordance with the experiments of Derksen *et al*. [55] that suggested that loss of *E-cadherin *could play a causal role in the acquisition of anoikis resistance, as parental mammary cells lacking E-cadherin survived while re-expression of the gene caused apoptosis [55]. Previous works show that loss of *E-cadherin *in either skin or mammary epithelium does not induce tumor formation [55]. Thus, an overall view of the events occurring in our HT29 cells resistant to methotrexate is needed.

It has been shown that activated PKCα translocates from the nucleus to the membrane [56], where it associates with caveolae [57,58], and regulates the function and formation of such biological structures. PKCα has been described to directly interact with CAV1. The union is performed between the caveolin 1 scaffolding domain peptide and PKCα caveolin 1 binding motif [59]. Further, activation of PKCα by phorbol esters dislocates the enzyme from caveolae. All these observations indicate that PKCα interacts functionally with this membrane structures. Moreover, PKCα has been proposed to be involved in the rearrangement of the cytoskeleton. Masur *et al*. showed that a high level of PKCα expression plus a low E-cadherin level predicts an elevated migratory activity of colon carcinoma cells, which could be derived more easily to metastasis [56]. Lahn *et al*. speculate that PKCα overexpression may represent an important cellular event leading to enhanced tumor progression, as they concluded that MCF-7 breast cancer cells transfected with PKCα had reduced expression of E-cadherin and β-catenin, resulting in a loss of cell-cell adhesion and thus in a more aggressive tumor phenotype [38].

**Figure 8:**
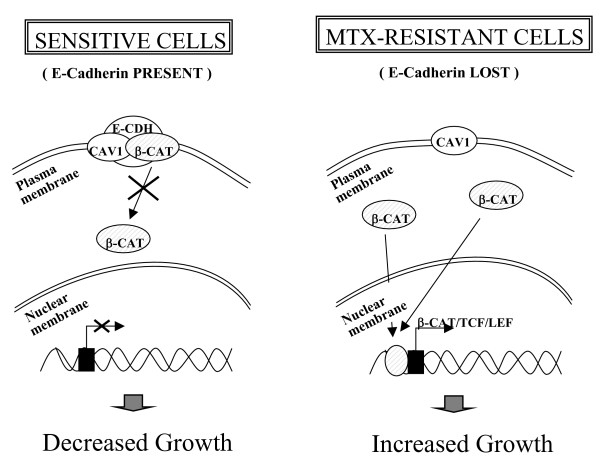
**Scheme for HT29 sensitive and MTX-resistant cells**. A diagram is presented showing the compartment localization of CAV1, E-cadherin (E-CDH) and β-catenin (β-CAT) in HT29 sensitive cells (with E-cadherin) and MTX-resistant cells (without E-cadherin, gene loss) and the effects caused by this differential situation. In the sensitive cells, β-catenin is located in the adherence junctions within a complex with E-cadherin. CAV1 co-localizes with β-catenin in these complexes, interfering in β-CAT signaling. If *E-cadherin *is lost, as in the resistant cells, β-catenin is not retained in the plasma membrane and then can be translocated into the nucleus, thus activating Tcf/Lef-1 transcription factors-mediated expression of genes implicated in cell proliferation and tumor progression.

Specific protein-protein interactions between CAV1 and other proteins have been proposed to regulate cell signaling [57,60]. Indeed, CAV1 is known to control cell proliferation and viability by inhibiting expression of survivin, a member of the IAP (inhibitor of apoptosis) family via a transcriptional mechanism involving the β-catenin-Tcf/Lef-1 pathway [16]. One of the possible locations of β-catenin is within a complex with E-cadherin in the adherence junctions, specialized cell-cell adhesion sites that link the cadherin molecules to the actin microfilaments [61]. E-cadherin promotes co-localization and co-imunoprecipitation of CAV1 with β-catenin, as well as inhibition of β-catenin-Tcf/Lef-1 dependent transcription of a wide variety of genes regulated by this pathway, among which survivin is found. However, the ability of CAV1 to regulate survivin expression and cell proliferation is severely impaired in metastasic cancer cells lacking E-cadherin [16]. If *E-cadherin *is lost, β-catenin is not retained in the plasma membrane and can be then translocated into the nucleus [62], thus activating Tcf/Lef-1 transcription factors-mediated expression of genes implicated in cell proliferation and tumor progression [50]. E-cadherin has been shown to be an important permissive element in defining the functions of CAV1, since several characteristics potentially relevant to CAV1 function as a tumor suppressor are compromised in E-cadherin-deficient HT29 cells [16]. A diagram showing all these relations is presented in Figure 8.

Our results show that HT29 cells can be well sensitized toward MTX by simultaneous treatment with siCAV1 and pBATEM2-CDH. Importantly, we can revert the resistant scenario by reducing the levels of caveolin 1 and by overexpressing E-cadherin simultaneously in the resistant cells, demonstrating the roles that play both genes in MTX resistance.

## Conclusion

We demonstrate that, aside from *dhfr*, the contribution of the 5q14 co-amplified genes to MTX resistance is small in HT29 colon cancer cells. On the other hand, we have identified genes deregulated in MTX resistant cells and have demonstrated a role for caveolin 1, E-cadherin, enolase 2 and PKCα in MTX resistance. Very importantly, the concomitant knocking down of CAV1 with overexpression of E-cadherin in HT29 resistant cells markedly reduced cell viability.

## Competing interests

The authors declare that they have no competing interests.

## Authors' contributions

ES carried out the siRNA and plasmid transfections, the determination of mRNA levels and associated cytotoxicity after these treatments, and drafted the manuscript. CM carried out the mRNA levels validations, the copy number determinations and helped to draft the manuscript. VN helped with data interpretation and to draft the manuscript, critically revising it. MAP participated in the design of the study and in its coordination, and helped to revise the manuscript. CJC conceived the study, participated in microarray data analyses and drafted the manuscript. All authors read and approved the final manuscript.

## Pre-publication history

The pre-publication history for this paper can be accessed here:



## Supplementary Material

Additional file 1 **Table of genes differentially expressed by 3 fold**. Excel file containing the list of 3-fold differentially expressed genes generated using GeneSpring software v 7.3.1. It includes the GenBank numbers of all genes, their respective common names and the associated description. The chromosomal localization of the 375 entries and the fold change values relative to the control are provided. A final column informing about one of the Gene Ontology categories to which the genes belong according to GeneSpring is included. The differentially expressed transcripts corresponding to open reading frames, transcribed sequences, cDNA clones or hypothetical genes were deleted. Click here for file

Additional file 2 **Primers used to validate mRNA levels of selected genes**. PDF file with sequences for the primers used to quantify the mRNA levels of genes studied, next to their common name and the number of the chromosome where they are located. Click here for file

Additional file 3 **Primers used to determine the copy-number of selected genes**. PDF file with common names, chromosome number and the sequences of primers used to amplify their respective DNAs for all selected genes. Click here for file

Additional file 4 **Sequences for the sense strand of all siRNAs used**. PDF file where the sequences for the sense strand of all the siRNAs used are provided next to the names used to designate all them and the genes they are directed against. Click here for file
