# Supplementary material for: Role of Caveolin 1, E-Cadherin, Enolase 2 and PKCalpha on resistance to methotrexate in human HT29 colon cancer cells
Source: BMC Med Genomics. 2008 Aug 11;1:35. doi: 10.1186/1755-8794-1-35 (PMC2527490; doi:10.1186/1755-8794-1-35)
Supplement: Additional file 2 — Primers used to validate mRNA levels of selected genes. PDF file with sequences for the primers used to quantify the mRNA levels of genes studied, next to their common name and the number of the chromosome where they are located. [file 1755-8794-1-35-S2.pdf]

Gene	Chromosome	Primers
S100A4	1	5'- ATGGCGTGCCCTCTGGAG -3'<br>5'- CCCAACCACATCAGAGGAGT -3'
ZFYVE16	5	5'- AATGGTACAAATAACTCCAGAG -3'<br>5'- CTTTAGAAAGTAGAACACCTCG -3'
DHFR	5	5'- ATCGGCAAGAACGGGGA -3'<br>5'- TCTGGAAAGAAAATGAGC -3'
MSH3	5	5'- CTTTCCAGGCAGTTACAG -3'<br>5'- CAAATCACAGGCTTCTCT -3'
RASGRF2	5	5'- AGGCAAGGATAGCAAACTG -3'<br>5'- GAAACATGGGATAGGGTCAAG -3'
XRCC4	5	5'- CAGAATCCACCTTGTTTCTGAAC -3'<br>5'- GTCATCAGCTTCTTGGGAAATC -3'
SSBP2	5	5'- CAAGAGGAATGGTGCCCTTAG -3'<br>5'- GACTAGGCATGATGGGTGTTC -3'
HAPLN1	5	5'- GGCTGATCATCTTTCAGACAA -3'<br>5'- CAGTGTAACATTGCCACCTCTG -3'
CAV1	7	5'- GCGACCCTAAACACCTC -3'<br>5'- AATAGACACGGCTGATGC -3'
AKR1C1	10	5' -GGTCACTTCATGCCTGTCCTG -3'<br>5' -GTCTTCTCTCTTCACACTGCCATC-3'
EN02	12	5'- TGCCTCAGAGTTTTATCGTG -3'<br>5'- CTTGAGCAGCAGACAGTTG -3'
CDH1	16	5'- AGGCCTCTACGGTTTCATAA -3'<br>5'- CTTGCCTTCTTTGTCTTTGTT -3'
PRKCA	17	5'- TATGGCGTCCTGTTGTATG -3'<br>5'- ACTTTGGGCTTGAATGGT -3'
18S	2	5'- GCGAAAGCATTTGCCAAGAA -3'<br>5'- CATCACAGACCTGTTATTGC -3'
PP1A	7	5'- CTCCTTTGAGCTGTTTGCAG -3'<br>5'- CACCACATGCTTGCCATCC -3'
