# Supplementary material for: Role of Caveolin 1, E-Cadherin, Enolase 2 and PKCalpha on resistance to methotrexate in human HT29 colon cancer cells
Source: BMC Med Genomics. 2008 Aug 11;1:35. doi: 10.1186/1755-8794-1-35 (PMC2527490; doi:10.1186/1755-8794-1-35)
Supplement: Additional file 3 — Primers used to determine the copy-number of selected genes. PDF file with common names, chromosome number and the sequences of primers used to amplify their respective DNAs for all selected genes. [file 1755-8794-1-35-S3.pdf]

Gene	Chromosome	Primers
S100A4	1	5'- CTTCCTGGGCTGCTTAT -3'<br>5'- ACTGGGCTTCTGTTTTCTATC -3'
ZFYVE16	3	5'- CATGCTGCCAACTAAAGAG -3'<br>5'- GAGCTATAAAGGGGAAAACAAT -3'
DHFR	5	5'- GGGGTTTTCCATAGTCA -3'<br>5'- GCCTCCAGTTTGCTTAC -3'
MSH3	5	5'- ATTTTAGAAGGGGTGGTG -3'<br>5'- TTAGGGGAAATTTAGATGCT -3'
RASGRF2	5	5'- ATTTTGATTGAGAGGGAATA -3'<br>5'- CAAGTTGATGTCGGAGTT -3'
SSBP2	5	5'- TGCACTCAATAGGGTAAGC -3'<br>5'- AAGACAGAGCGTAAGGTAACA -3'
XRCC4	5	5'- AATCCCATGTCTCTTCTCC -3'<br>5'- TTTAATGCCAATCCTCTCC -3'
HAPLN1	5	5'- GGCTGATCATCTTTCAGACAAC -3'<br>5'- CTGCACTTAACAGCCTTGAAC -3'
EDIL3	5	5'- CTTCCCACAGTCCTTTTC -3'<br>5'- AATTTATTGCCAGCTATCTTTC -3'
PSMB8	6	5'- GCACCCACCTTCTTATCC -3'<br>5'- GGCTGCCTTTGTATGTTTC -3'
VNN1	6	5'- CAGCCACTCAGCTCCTCAGC -3'<br>5'- GAGTCCCTGTTGAAGTTCCAG -3'
CAV1	7	5'- CCCCCAATAGTTAGTTCAG -3'<br>5'- CTTCCCATCGTTTTAGTT -3'
MTUS1	8	5'- TAATGGGATGAATGACACC -3'<br>5'- CCCCCTGAGAAAACACT -3'
AKR1C1	10	5' -GGTCACTTCATGCCTGTCCTG -3'<br>5' -CTGTGACAAGATGGCATTGCAG -3'
ENO2	12	5'- CATCCCTCCTGCTTGTA -3'<br>5'- AGTCATATTTGCCATCACG -3
DHRS2	14	5'- CTGTTGCTGGCCTTGTCTGTC -3'<br>5'- GGTGTCTGAGATGCTTCCTGC -3'
CHD1	16	5'- TATGGTTTGTTTTGGTTGTG -3'<br>5'- AAATGGATCTGTGGGTTATG -3'
PRKCA	17	5'- AGTTTCCTTTTTCTCTACCA -3'<br>5'- ATTTCAGGACTACTTACATCAG -3'
SLC19A1	21	5'- TCTCAGCATTCAGTCTTCAC -3'<br>5'- ATCACCTATGTTCCTTTGTCA -3'
IRAK1	X	5'- CCGGAAGTTGCCATCCTCAG -3'<br>5'- GACCAGCAGCAGGGTCTACAG -3'
IDH3G	X	5'- GCAGGTTATGGTTGGTTTC -3'<br>5'- ACTTGTTCATTCCGTCGTT -3'
ATP6AP1	X	5'- GAGGTGGACTGTTCCTTGCTC -3'<br>5'- CTGCAAGTCGCTGGTGATGTG -3'
B2M	15	5'- CCAAGTCACGGTTTATTCT -3'<br>5'- TATTGCCAGGGTATTTCA -3'
