# Supplementary material for: Role of Caveolin 1, E-Cadherin, Enolase 2 and PKCalpha on resistance to methotrexate in human HT29 colon cancer cells
Source: BMC Med Genomics. 2008 Aug 11;1:35. doi: 10.1186/1755-8794-1-35 (PMC2527490; doi:10.1186/1755-8794-1-35)
Supplement: Additional file 4 — Sequences for the sense strand of all siRNAs used. PDF file where the sequences for the sense strand of all the siRNAs used are provided next to the names used to designate all them and the genes they are directed against. [file 1755-8794-1-35-S4.pdf]

Gene	siRNA name	siRNA sequence
ZFYVE16	siZFYVE16	5'- GGATCACGGAGGATTCCTGTT -3'
DHFR	siDHFR	5'- AATGAGCTCCTTGTGGAGG -3'
MSH3	siMSH3	5'- GCAACATCACAGTCCTTGGTT -3'
SSBP2	siSSBP2	5'- AACCACAGTGAGTCAGCCCTT -3'
XRCC4	siXRCC4	5'- CTGATCTCTCTGGGTTGGCTT -3'
HAPLN1	siHAPLN1	5'- GTGTGAGGTGATTGAAGG -3'
EDIL3	siEDIL3	5'- GTGAAGCATACCGAGGGGATACATT -3'
ENO2	siENO2	5'- CTGCCCCTGTATCGCCACA -3'
PRKCα	siPRKCα	5'- TCCGCAGTGGAATGAGTCC -3
CAV1	siCAV1	5'- CCAGAAGGGACACACAGTT -3'
-	NR-siRNA	5'- ATGTGGAAGCAGGAGAGAT -3'
